# Intra-tumor heterogeneity from a cancer stem cell perspective

**DOI:** 10.1186/s12943-017-0600-4

**Published:** 2017-02-16

**Authors:** Pramudita R. Prasetyanti, Jan Paul Medema

**Affiliations:** 1 Laboratory for Experimental Oncology and Radiobiology (LEXOR), Center for Experimental Molecular Medicine (CEMM), Academic Medical Center (AMC), University of Amsterdam, 1105AZ Amsterdam, The Netherlands; 2 Cancer Center Amsterdam and Cancer Genomics Center, Amsterdam, The Netherlands; 3 Academic Medical Center, Meibergdreef 9, 1105AZ Amsterdam, The Netherlands

**Keywords:** Tumor heterogeneity, Cancer stem cell, Stemness, Tumor microenvironment

## Abstract

Tumor heterogeneity represents an ongoing challenge in the field of cancer therapy. Heterogeneity is evident between cancers from different patients (inter-tumor heterogeneity) and within a single tumor (intra-tumor heterogeneity). The latter includes phenotypic diversity such as cell surface markers, (epi)genetic abnormality, growth rate, apoptosis and other hallmarks of cancer that eventually drive disease progression and treatment failure. Cancer stem cells (CSCs) have been put forward to be one of the determining factors that contribute to intra-tumor heterogeneity. However, recent findings have shown that the stem-like state in a given tumor cell is a plastic quality. A corollary to this view is that stemness traits can be acquired via (epi)genetic modification and/or interaction with the tumor microenvironment (TME). Here we discuss factors contributing to this CSC heterogeneity and the potential implications for cancer therapy.

## Background

A tumor is a heterogeneous population of cells, containing transformed cancer cells, supportive cells and tumor-infiltrating cells. This intra-tumor heterogeneity is further enhanced by clonal variation and microenvironmental influences on cancer cells, which also do not represent a homogeneous set of cells. Early observations showed that tumors contain subclones that differ in respect to karyotype and sensitivity to chemotherapy [1, 2]. More recent profiling endeavours, using in-depth sequencing and methylation profiling of various tumor regions, revealed multiple clones with both distinct genetic mutations and promoter hypermethylation within a single tumor [3, 4]. Importantly, the nature of this heterogeneity is not limited to the malignant cancer cell population only as a tumor is a complex ecosystem containing tumor cells and other cell types, such as endothelial cells, infiltrating immune cells, stromal cells as well as a complex network of extracellular matrix (ECM), which defines spatiotemporal differences in the tumor microenvironment [5, 6]. Conceivably, both tumor and microenvironment heterogeneity determine the fitness of the tumor and as such, are likely to be crucial factors in treatment success.

Two models have been proposed to account for heterogeneity within a tumor. In the clonal evolution model, stochastic mutations in individual tumor cells serve as a platform for adaptation and selection for the fittest clones of a tumor. As such, this model explains intra-tumor heterogeneity as a result of natural selection. The clones that acquire growth advantage will expand while the clones with less fitness will be competed out and may eventually become extinct. Importantly, such clonal advantages may differ in time and space as different requirements may be present in different areas of the tumor. Certain areas may select for “hypoxia-fit” clones, while other more nutrient dense regions may select for fast-growing clones. During the course of the disease, these clones may change spatially and temporally resulting in a complex sub-clonal architecture, which is further enhanced by the application of therapy [7–9]. The second model that is proposed to install intra-tumor heterogeneity is the cancer stem cell (CSC) model. This model suggests that only a subset of cancer cells possess indefinite self-renewal ability to initiate and maintain tumor growth. Therefore, tumors are organized in a hierarchical fashion, equivalent to the normal tissue hierarchy supported by healthy stem cells. Accordingly, CSCs generate cellular heterogeneity by installing a differentiation hierarchy leading to a range of distinct cell types present within the tumor [10]. It should be noted however, that this hierarchy is not a one-way route, but can be reversible or plastic whereby the terminally differentiated cells can also dedifferentiate and gain CSCs properties under specific conditions [11, 12]. The concept of cell plasticity has partly reconciled both stochastic and CSC models. For instance, mutation in a differentiated cell can endow self-renewal capacity and establish a new hierarchical CSC clone, adding the functional diversity within a tumor [13, 14].

Below we provide the overview on how stemness features are installed in (cancer) cells and hence, influence plasticity of this population. We first zoom in on intrinsic factors, like genetic and epigenetic factors, which we consider to be the inherent properties contributing to self-renewal capacities. Secondly, we will discuss extrinsic factors, like the tumor microenvironment and therapy, that can influence cellular phenotypes. Exploring the mechanism of self-renewal and plasticity competency may allow researchers to interfere with these processes and ultimately, improve cancer management.

## Main text

### CSC model

The concept of cancer stem cells was first formally tested in hematological malignancies. Lapidot and co-workers showed that the CD34^+^/CD38^−^ subpopulation from acute myeloid leukemia (AML) was able to form leukemia after transplantation into NOD/SCID mice [15]. Since this seminal publication, cell purification using distinct surface markers followed by transplantation in immunocompromised mice has been used as gold standard to identify functional CSC populations. With this method, CSCs can be purified from diverse types of haematological and solid malignancies such as breast, glioma, colon, pancreas and liver [11, 16]. These efforts, however, were faced with a strong scepticism, as the purification of CSCs requires dissociation of human tumor material into a single-cell suspension followed by transplantation in immune-deficient mice. This procedure releases cancer cells from their natural environment and exposes them to a hostile new environment, which may change their behaviour. Hence, it is unclear whether the purified cells will also function as CSCs in an intact tumor setting and importantly, whether the nature of such CSCs is clinically relevant. The first clear evidence to support a role for CSC activity in intact tumors is provided by three independent studies in brain, skin and intestinal tumor mouse models. Using the genetically engineered lineage-tracing technologies, these studies provided clear evidence that CSCs arise *de novo* and drive tumor growth [17–19]. These studies seem to resolve the debate whether CSCs do exist or are merely a xenotransplantation artefact. However, formally these studies do not exclude the possibility that more differentiated cells can also fuel cancer growth, potentially under conditions of stress or specific therapy. Although one of these studies did reveal that CSCs were essential for repopulation of the tumor after drug treatment and that this could be prevented by the addition of a CSC-specific drug [19]. Similarly, targeting of intestinal CSCs using LGR5 antibodies displayed a dependency on CSCs for tumor survival [20]. In addition, a handful of preclinical and clinical observations demonstrated that CSCs selectively resist therapy and can be responsible for tumor relapse [21], suggesting that eradication of a cancer would require killing of CSCs. Nevertheless, the key question is whether targeting of CSCs alone is sufficient or whether non-CSCs could take their place after de-differentiation.

**Fig. 1:**
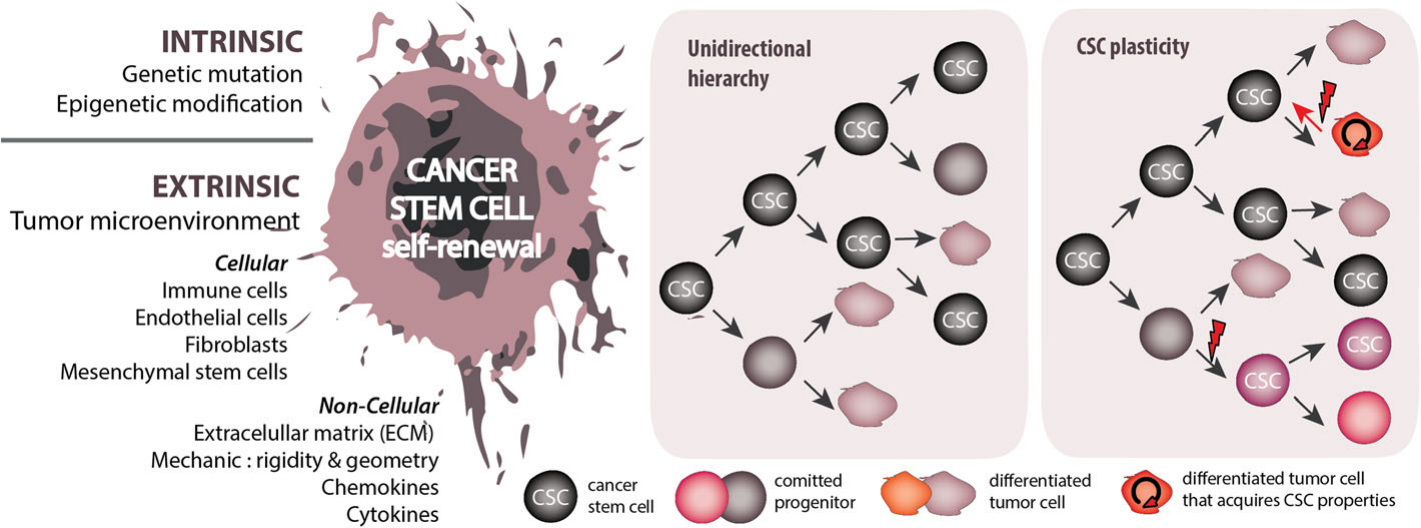
The original CSC model (unidirectional hierarchy) assumes that only CSCs are able to generate the bulk of tumor via symmetric division (to self-renew) or asymmetric division (to generate differentiated cells). In this case, the hierarchy is strictly unidirectional and precludes the concept of cell fate reversibility from the progenitor cells. In contrast, accumulating evidence demonstrate that the hierarchy is more fluid than originally thought. In the CSCs plasticity model, (cancer) cell posses the dynamic ability of bidirectional conversion from a non-CSC state to a CSC state and vice versa. In this model, the stemness and CSCs plasticity are determined by diverse intrinsic and extrinsic cues that work simultaneously or independently overtime. Consequently, non-CSCs can serve as reservoir to create CSC populations throughout tumorigenesis. In the figure this is indicated with a lightning bolt and can be the result of a microenvironmental cue or a (epi-)genetic change

Unfortunately, the efficacy of CSC targeting and the capacity to revert to the CSC state has been difficult to study due to the limited characterization of CSC markers. Several markers, such as CD133, CD44, CD166, CD24, and ALDH1 activity, have proven useful for prospective isolation of CSCs in multiple solid tumors [11]. However, CSC marker expression is not uniform between tumor types. For instance, while CD133 has been used as a marker to identify CSCs in glioblastoma [22] and CRC [23], it is not a reliable marker in breast cancer where CD44^+^CD24^−^ is commonly used to enrich for CSCs [24]. CSC markers expression also varies between cancer subtypes and even, between patients in the same subtype [16]. For instance, CD44^high^CD24^low^ fails to efficiently enrich CSCs in triple negative breast cancer [25] and CD133 has been debated in colon cancer. Furthermore, the lack of consistency has generated confusion in the field of CSC identification and questioned the functionality of CSC markers [26–28]. A possible explanation could be that purified populations may remain heterogeneous and may require additional markers to allow optimal CSC enrichment. Indeed, the combination of CD44, EpCam and CD166 could identify CSCs in CRC more robustly than CD133 alone [29]. Adding another layer of complexity, the genetic and epigenetic changes influence CD133 surface marker expression as well as modify the detection with the commonly used antibodies [30, 31]. Consequently, the absence of CD133 expression may actually reflect the detection limit and give a false-negative rate in identifying CSCs. These observations indicate that the phenotype of CSCs is not as well defined as would be required for optimal detection in clinical material. Instead, CSC markers can be viewed as a property of cells that is highly context dependent. Furthermore, accumulating evidence suggest that self-renewal traits of CSCs can be acquired and dynamic rather than fixed in a defined cell population. In this concept, the CSC model is not necessarily rigid and unidirectional as non-CSCs can regain CSC characteristics depending on various intrinsic and extrinsic factors. These factors influence stemness properties and thereby contribute to the functional diversity of a single tumor (Fig. 1).

### Intrinsic features: genetic and epigenetic

Cancer arises through accumulation of mutations that install a malignant phenotype [32]. As neoplastic lesions develop, mutant clones expand and are subjected to further (epi)genetic alterations and microenvironmental pressure [33] resulting in clones that have acquired the different “hallmarks of cancer” [34]. Whether these oncogenic mutations are required to occur in specific cell populations, such as stem cells or progenitor cells, remains a subject of debate. The propensity of cells to undergo transformation and initiate tumorigenesis could be either a stochastic process or be predefined by the cell of origin (stem cell vs non stem cell compartment). It is plausible that CSCs originate from normal stem cells and exploit the molecular machinery already present in these healthy stem cells, such as self-renewal and tissue regeneration, to perpetuate indefinitely [35]. A contemporary mathematical model supports this view by demonstrating a nearly perfect correlation between cancer risk and the rate of stem cell division, suggesting highly replicative stem cells as alleged target for mutation and hence, neoplastic transformation [36]. Recently an elegant study by Zhu and colleagues provided direct evidence that mutations in stem cells dictate cancer risk. Using lineage tracing of CD133^+^ cells, they showed that stem cells, particularly in adult tissue, were inherently susceptible to neoplastic transformation and produced tumors upon activation of oncogenic mutations [37]. Such oncogenic transformation of stem cells can cause disturbance in cell division or a block in differentiation leading to stem cell expansion. For example, introduction of NRAS(G12D) in normal hematopoietic stem cells (HSC) reprogramed transcriptional response and cell-cycle kinetics. This signal alone increased the proliferation and resulted in a clonal advantage over normal HSC in serial transplantation assays [38]. Furthermore, transformed stem cells highly expressed genes for immune regulators, such as CTLA4 and CD274 (PD-L1) [37]. This observation suggests that in the earliest stage of tumorigenesis, transformed stem cells not only propagate mutations, but importantly also install a protection of the tumor from immunosurveillance.

While it may be intuitive that CSCs originate from transformation of healthy stem cells, several studies have pointed out that stem cells and differentiated cells represent an equally permissive pool for tumorigenesis (reviewed in [11]). An initial report suggested that oncogene expression in terminally differentiated cortical astrocytes and neurons initiated glioblastoma [39]. The genetically acquired plasticity drives cancer progression and is even able to facilitate transdifferentiation into blood vessels, further sustaining the malignancy [40]. Similarly, specific dysregulation of signaling pathways in differentiated cells can also dictate the emergence of neoplastic cells. For instance, in a mouse model for intestinal tumor formation, aberration of Wnt and NFkB pathways in non-stem cells initiated tumorigenesis [41]. The above describes how CSCs can be induced by genetic perturbation. It is however important to realize that the CSC hierarchy in cancers also appears to be more fluid than originally thought. That is, under the right genetic or epigenetic alterations, non-CSCs can dedifferentiate and acquire CSC features.

Although proof for plasticity in both healthy and cancerous tissue has accumulated tremendously in recent years, the knowledge on how this plasticity is orchestrated is still in its infancy. The different models indicate that genetic perturbations can play a prominent role in installing self-renewal capacity, but genetic change alone is not sufficient to induce all phenotypes. It is clear that cancer initiation and progression induced by oncogenic mutations are accompanied by significant epigenetic alterations as well, including genome wide changes in DNA methylation (hypomethylation), CpG islands promoter hypermethylation, histone modification patterns and nucleosome remodelling [42]. Genetic and epigenetic alterations can be considered two sides of the same coin. Both processes are intertwined and benefit from each other in driving tumorigenesis. As such, alterations in the epigenome can lead to mutations, while mutation of epigenetic regulators can induce epigenetic chain reactions. For instance, promoter methylation of critical genes, such as DNA repair genes, may predispose normal cells to genetic lesions. A clear example of this is silencing of mismatch repair genes causing an accumulation of mutations and instability of microsatellites [43]. Alternatively, epigenetic alterations can deregulate fundamental signalling pathways controlling self-renewal and differentiation, including Wnt, Notch, Myc and Hedgehog pathways (reviewed in [44]). An example of such pro-tumorigenic event is the silencing of Wnt inhibitors, which leads to proliferative advantages that may expand the pool of cells that are eligible for oncogenic mutation and thereby increases cancer risk [45]. Conversely, there is emerging evidence that genetic mutations may also directly lead to epigenetic alterations that control cellular fate. One meaningful example is provided by a recent study on the role of DNA methyltransferase 3A (DNMT3a) mutation in hematological malignancy, which cooperates with RAS mutation to produce AML [46]. Although RAS mutation alone induces hyper-proliferation, it is not sufficient to support self-renewal and induce malignancy [47]. DNMT3a mutations occur frequently in AML. Mechanistically, mutated DNMT3a activates distinct enhancers to induce focal DNA methylation and histone acetylation leading to deregulations of stemness pathways. Especially the Meis1-Mn1-Hoxa gene clusters are shown to be critical for DNMT3a mediated AML progression. As a result, DNMT3a mutation can confer aberrant self-renewal and block differentiation, but is not sufficient to induce hyper-proliferation. Combination of DNMT3a and RAS mutation therefore results in a highly penetrant AML and exemplifies the synergism between genetic and epigenetic alteration in initiating a self-renewing proliferative CSC population and thereby malignancy [46]

Next to a role in the onset of cancer, it has been proposed that epigenetic modifications dictate the phenotype of CSCs in established tumors. An example of how epigenetic plays a role in modulating CSC properties is represented by the epithelial-mesenchymal transition (EMT) process. Studies in breast cancer link EMT with acquisition of CSC features, such as the expression of surface markers associated with breast CSCs (CD44^high^CD24^low^) and increased self-renewal plus tumor initiating capacity [48–50]. Recent studies provide clear cues that EMT relies on various epigenetic modifications that impact on the expression of the mesenchymal transcription factor ZEB1, providing a direct link between epigenetics and CSCs [51, 52]. Unlike gene mutation that can affect gene expression in a straightforward manner, stable epigenetic marks may require a complex fine-tuning modification of chromatin. For example, certain gene promoters can contain both a permissive (H3K4me3) and a repressive histone mark (H3K27me3). The co-existence of both antagonistic marks has been referred as ‘bivalent chromatin’ and can be found in many developmental regulatory genes [53, 54]. Ultimately, the genes with bivalent state are poised for transcriptional activation or silencing upon the correct incoming cues [55]. In the case of breast cancer, CD44^low^ subpopulation maintain the ZEB1 promoter in the bivalent state, which allow it to be activated into an active chromatin configuration upon stimulation with transforming growth factor-beta (TGF-β). Consequently, ZEB1 transcription increases and CD44^low^ cells convert into CD44^high^ cells along with the acquisition of CSC functional traits [51]. In another example, hypoxia is shown to induce EMT via an epigenetic mechanism that involves inhibition of oxygen-dependent H3K27me3 demethylases, which results in silencing of the *DICER* promoter, the enzyme involved in microRNA processing. This leads to decreased production of miRNAs of the mir200 family and subsequently, de-repression of mir200 family target including ZEB1. As a result, ZEB1 expression increases and eventually leads to the acquisition of a CSCs phenotype [52]. Taken together, genetic and epigenetic alterations are deterministic in the establishment of stemness traits. Importantly, there is a growing body of evidence pointing out that a favourable environment is essential in the dedifferentiation of tumor cells into CSCs. Further identification of more detailed microenvironmental signals that support or determine the stemness is of paramount importance to allow for better intervention strategies.

### Extrinsic features: the tumor microenvironment

Tumor cells are under constant selection pressure, which is a result of the changing conditions within the microenvironment or due to applied therapy. From a CSC perspective there are several possible mechanisms by which cancer therapy can change tumor intra-heterogeneity. First, therapy acts as selection mechanism that shapes tumor evolution. As CSCs are thought to be inherently (more) refractory to chemotherapy, this population can be selected for upon therapy, changing the intra-tumor heterogeneity [21]. However, within the CSC population there is room for clonal variation as well, i.e. distinct CSC-driven clones that differ in their growth speed or therapy resistance. Consistent with therapy acting as a selective force, chemotherapy resulted in the outgrowth of slowly proliferating cell populations and/or previously dormant CRC clones [56]. In addition, clonal diversity was shown to be reduced in breast cancer [57, 58], suggesting that intra-tumor heterogeneity is changed, mostly reduced, upon therapy. A second means by which therapy can change intra-tumor heterogeneity is by inducing phenotypic plasticity. For instance, it has been reported that therapy induces *de novo* generation of cells with CSC properties. For example, study in breast cancer demonstrated that taxane induces transition of differentiated cells into a CSC state (CD44^high^CD24^high^) and further contributed to the therapeutic resistance [59].

**Fig. 2:**
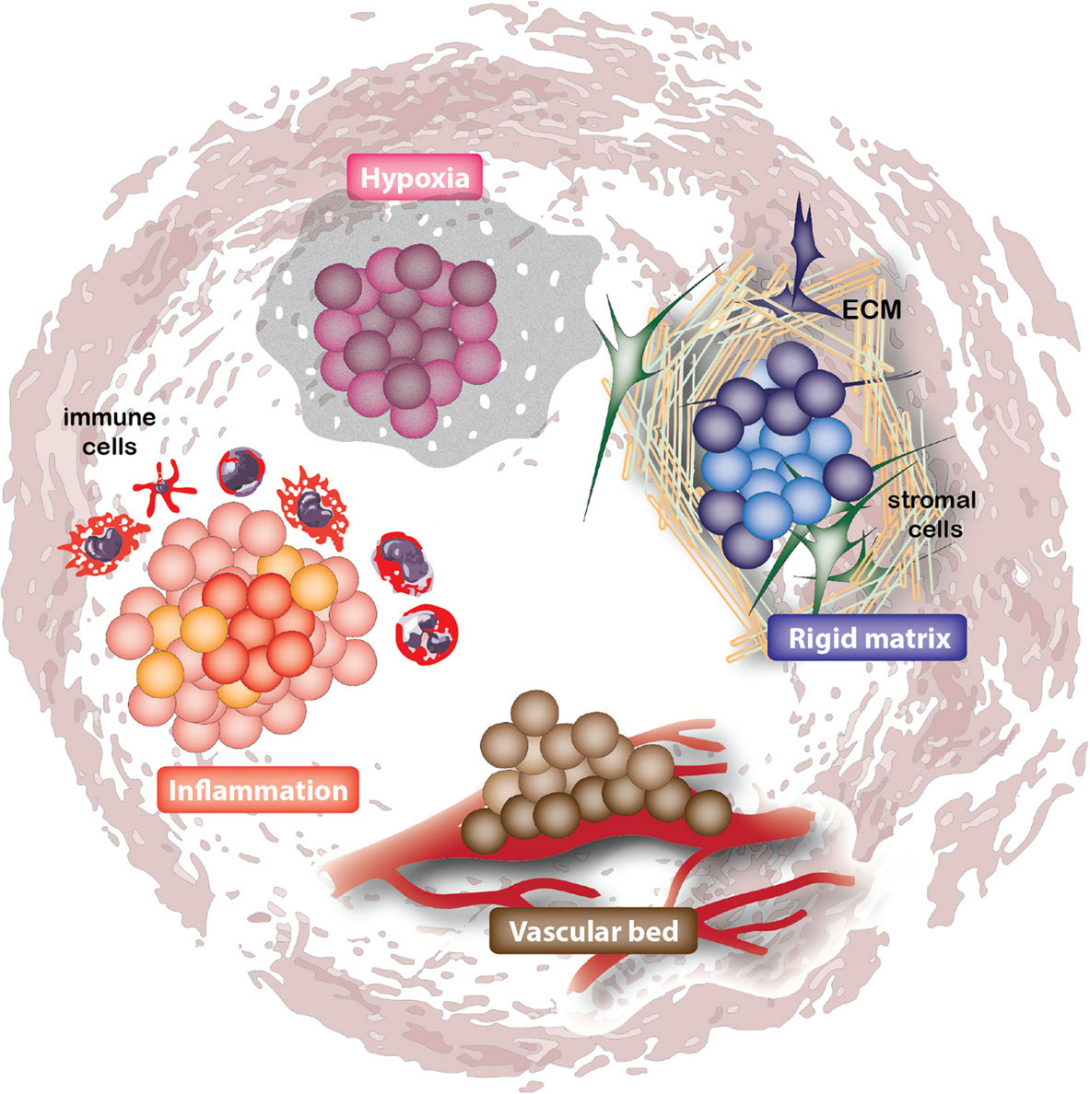
Next to intrinsic factors, the tumor microenvironment plays a crucial role in influencing cell state. The tumor microenvironment, in addition to hosting the tumor cells, possess a dynamic topography within the tumor involving diverse supportive ECM scaffolds, growth factors, a vascular bed and immune cell interactions [6]. The right combination of microenvironment components, for example inflammation, hypoxia, vascularized niche or rigid matrix, potentially contribute to stemness and enhanced tumorigenicity [52, 62, 68, 87, 91]. Multiple (distinct) niches may co-exist within a tumor, leading to cellular diversity

The role of the microenvironment in this selection process and (Fig. 2) on fate determination and the behaviour of cells is considered to be major [60]. A clear example as to how the microenvironment can influence cancer initiation is shown by the chronic inflammation induced by Helicobacter pylori which is strongly is linked to increased risk of developing stomach cancer. Similarly, patients with inflammatory bowel disease (IBD) have an associated increased risk for colon cancer [61]. Indeed, an inflammatory microenvironment has been suggested to induce proliferation of pre-cancerous lesions, thereby facilitating tumorigenesis [62]. However, the mechanism as to how inflammatory signals exacerbate tumor development is poorly understood. More recently, it is shown that induction of mutations in CD133^+^ cells in normal adult liver does not lead to tumor formation unless local tissue damage is induced [37], leading to a speculative model in which an inflammatory environment provides an advantage to mutated stem cells. In agreement, it was shown that intestinal stem cells with a p53 mutation do not have a competitive advantage over untransformed stem cells under normal conditions, but in the presence of inflammation outcompete their normal neighbors likely facilitating further tumorigenesis to occur [63]. Therefore, the combined effects of genetic lesions in (stem) cells with epigenetic alterations and microenvironment components can initiate tumor development by favoring a competitive advantage for the transformed (cancer) stem cell.

How then does the microenvironment stimulate stem cell expansion? In the case of inflammation, immune cells release a range of inflammatory cytokines, such as interleukin (IL)-1, IL-6, and IL-8 [60]. These all activate Stat3/NF-κB in both stromal and tumor cells, creating a positive feedback loop to maintain a chronic inflammatory state in tumor cells. These cytokines, particularly IL-6, have been shown to cause differentiated tumor cells to dedifferentiate into CSCs [64]. Next to inflammatory mediators, the tumor microenvironment is known to direct tumor growth in other ways. The unique composition of the microenvironment, both in terms of the extra cellular matrix (ECM) and the cells surrounding the cancer cells, such as cancer associated fibroblasts (CAF), endothelial and immune cells, plays an important role in tumor maintenance. Stromal cells have been reported to mediated paracrine signaling, which can modulate the CSC phenotype. For instance, high expression of nuclear β-catenin, which is associated with active Wnt signaling and defines the colon CSCs, is detected within the colon cancer cells that reside close to stromal myofibroblasts. In fact, we reported that hepatocytes growth factor (HGF) secreted by myofibroblasts can facilitate Wnt signaling, which is not only important for CSC maintenance but can also induce de-differentiation of non-CSCs into CSCs [65]. Microvasculature surrounding the tumor is another relevant example of a microenvironment component that supports cancer growth. Many studies have proposed that vasculature could provide a specialized niche for CSCs, since leukemic, brain, colon and skin CSCs are often found to reside next to a vascular bed [34]. Subsequent discoveries supported this model and showed that endothelial cells promote CSCs properties. For instance, endothelial cells have been shown to induce a CSC phenotype in colon cancer via production of Notch-ligand DLL4 [66]. In line with this finding, our group showed that secreted growth factors from endothelial cells support and induce stem cells features in glioblastoma [67, 68]. Apparently, tumor cells hijack the normal tissue machinery and utilize growth factor present in the tumor microenvironment. In several cases this is an active process where tumor cells either instruct the microenvironment attracting for instance CAFs [69] or endothelial cells through VEGF secretion [70]. Alternatively, CSCs can even create their own niche via transdifferentiation into for instance endothelial progenitor cells [71, 72], which then provide essential growth factors to the CSC population. Intriguingly, this later process was not prevented by administration of angiogenesis inhibitors [70, 71]. A better insight into this mechanism may thus provide a potential novel approach to eradicate such tumors.

Another aspect of the microenvironment that possesses the power to influence cancer cell behavior is the ECM [73]. For instance, slight changes in matrix composition affect the phenotype of breast cancer [74, 75]. The ECM exerts its effect through so called mechano-transduction. Differential matrix stiffness and geometry are transmitted through cell-matrix contact and cell-to-cell-adhesion sites. Changes in mechanical forces are rapidly detected by the cellular cytoskeleton, creating tension within the cytoskeleton. Subsequently, cells respond to such mechanical stimuli by changing their shape and behavior [76]. Changes in the ECM have been shown to precede tumor development, favor neoplastic growth and contribute to metastasis [77, 78]. For instance, increased collagen content in the ECM enhances mammary tumor formation [79]. More recently, YAP/TAZ, transcriptional co-activators of the Hippo pathway, were shown to function as sensor and mediator of ECM mechanical cues [80]. In cancerous tissue, YAP/TAZ activity is increased specifically within tissue regions exhibiting higher collagen crosslinking [81]. Importantly, the role of YAP/TAZ in sustaining CSC features has emerged in several cancer types [82]. TAZ has been shown to install self-renewal capacity in non-CSCs and expands the pool of CSCs [83]. Similarly, YAP expression marks CSCs and maintains CSCs features through SOX2-Hippo signaling pathway [84]. Combined these observations suggest a direct role for the ECM in CSC maintenance through the activation of YAP/TAZ. Next to YAP/TAZ, Integrin-linked kinase (ILK) has recently emerged as a key actor of the cell-ECM cross-talk. Its expression has been associated with advanced tumor [85, 86] and through its interaction with β1-integrin, ILK responds to matrix stiffness activating an ILK/PI3K/Akt pathway, leading to up-regulation of self-renewal capacity in CSCs. This activation is further increased by hypoxic microenvironment [87]. Altogether, mechanical signals and physical features from the microenvironment influence many fundamental traits of CSCs. Future work on means to manipulate the mechano-stimuli from ECM, either through genetic perturbations or carefully designed experimental approaches are therefore crucial to provide new insights in CSCs biology.

## Conclusion and perspective

Cancer is an exceptionally complex and robust disease. The diverse genetic and epigenetic alterations, along with the interaction between cancer and the surrounding microenvironment mark the tumor heterogeneity. In this review, we discussed various features that install self-renewal in CSCs and how CSC plasticity fuels intra-tumor heterogeneity. Delineating features surrounding these processes will enable researchers to understand the complex signaling mechanisms that underlie the CSC state. Although we have come to understand important aspects of CSC biology there is still a tremendous gap in our knowledge, particularly in how we can optimally model the nature of the tumor microenvironment, including the three-dimensional (3D) cell-to-cell contact, cell-matrix contact and the multi-cellular components, such as stromal and immune cells. So far, researchers have traditionally relied on the use of two-dimensional (2D) cancer cell line as a source to model cancer. The failure to capture components of the microenvironment in this model has been perceived as a determining factor for the disappointing success rate of novel drugs in oncology [88]. The recent switch to primary patient-derived cancer material and the development of 3D culture with the use of Matrigel® has significantly improved such models and shown to better recapitulate intra-tumor heterogeneity [89]. Despite a poorly defined composition, this matrix has shed tremendous useful insight on tumor biology and enabled high throughput screening [90]. However, despite a clear improvement, current 3D cultures normally do not include supportive cells normally present in the tumor. In addition, the matrix composition and rigidity are not the same as in cancer. With this in mind, the future development of cancer models ideally should accommodate the heterogeneous components of a tumor. For instance, co-culture of patient’s own cancer and stromal cells in specialized scaffolds representing ECM physical features will definitely open up novel insight into CSC biology and may provide crucial insight to develop CSC-specific therapies.

## Abbreviations

2D: Two-dimensional
3D: Three-dimensional
ALDH1: Aldehyde dehydrogenase isoform 1
AML: Acute myeloid leukemia
CAF: Cancer associated fibroblast
CRC: Colorectal cancer
CSC: Cancer stem cell
ECM: Extra cellular matrix
EMT: Epithelial mesenchymal transition
HSC: Hematopoietic stem cell
IL: Interleukin
